# Enhanced Deposition Uniformity via an Auxiliary Electrode in Massive Electrospinning

**DOI:** 10.3390/nano6070135

**Published:** 2016-07-22

**Authors:** Dezhi Wu, Zhiming Xiao, Lei Deng, Yu Sun, Qiulin Tan, Linxi Dong, Shaohua Huang, Rui Zhu, Yifang Liu, Wanxi Zheng, Yang Zhao, Lingyun Wang, Daoheng Sun

**Affiliations:** 1 School of Aerospace Engineering, Xiamen University, Xiamen 361005, China; xiaozhiming@stu.xmu.edu.cn (Z.X.); dengl@stu.xmu.edu.cn (L.D.); xmusunyu@stu.xmu.edu.cn (Y.S.); huangshaohua27 @stu.xmu.edu.cn (S.H.); zhurui@xmu.edu.cn (R.Z.); yfliu@xmu.edu.cn (Y.L.); zhaoy@xmu.edu.cn (Y.Z.); sundh@xmu.edu.cn (D.S.); 2 Science and Technology on Electronic Test and Measurement Laboratory, North University of China, Tai Yuan 030051, China; tanqiulin.99@163.com; 3 Key Laboratory of RF Circuits and System of Ministry of Education, Hangzhou Dianzi University, Hangzhou 310018, China; donglinxi@hdu.edu.cn; 4 Department of Mechanical Engineering, University of California, Berkeley, CA 94720, USA; 5 Changsha Tobacco Monopoly Administration, Changsha 410007, China; monkeyxihao@sina.com; 6 State Key Laboratory of Luminescent Materials and Devices, South China University of Technology, Guangzhou 510640, China

**Keywords:** electrospinning, auxiliary electrode, deposition uniformity

## Abstract

Uniform deposition of nanofibers in the massive electrospinning process is critical in the industrial applications of nanofibers. Tip-Induced Electrospinning (TIE) is a cost-effective large-scale nanofiber-manufacturing method, but it has poor deposition uniformity. An auxiliary conductive electrode connected to the emitting electrode was introduced to improve the deposition uniformity of the nanofibers. The effects of the auxiliary electrode shape, the tilted angles and the position of the boat-like electrode on the electric field distribution, the diameter of the nanofibers, the jet control and the deposition uniformity were explored by using finite element analysis of the electric field and experiments. Experiments showed that the boat-like electrode at 20 mm above the reservoir bottom with a 5° tilted angle helped to decrease the relative deposition error of nanofibers in the greatest extent to about 5.66%, indicating such an auxiliary electrode is a good candidate method to greatly improve the deposition uniformity of nanofibers in massive electrospinning.

## 1. Introduction

Electrospinning is a bottom-up, facile, versatile and pure mechanical stretching technique to fabricate nanofibers and it has garnered much attention for about 20 years. Due to the excellent properties of high surface area to volume and high porosity, electrospun nanofibers can be widely applied in filtration [1], separators for batteries [2,3], energy harvesting [4,5], sensors [6] and so on. Many literatures about massive electrospinning have been reported to improve nanofiber productivity and great progress has been achieved [7,8,9,10,11]. Naturally, multiple spinnerets electrospinning with different arrangements have been explored and it was found that the jets are greatly affected by the changed electric field [12]. The electric field interference between the adjacent spinnerets would lead to a clog problem, while needle-less electrospinning can avoid such a problem. Elmarco used moving metallic wires as emitting electrodes to realize continuous production and has developed a series of the commercial setup Nanospider^™^ based on the patent technology [13]. The wires are parallel and their gap distance is set to be several centimeters. Our previous work proposed a method called Tip-Induced Electrospinning, where a tips array is utilized to dip into the polymer solution surface and draw away periodically to induce the generation of Taylor cones therein, and then multi-jets issue from the vertexes of the Taylor cones to gain a productivity of about 2 *g*/*h* from a solution surface area of 5 cm × 12 cm [14]. It is characterized to be of lower driving voltage threshold, easy scale-up and high throughput for each jet. However, the deposition uniformity of the nanofiber membrane is poor because many jets travel randomly to the collector and some jets even get to the supporting platform. Thus, it inhibits their industrial applications that need good thickness uniformity, for example separators for Li-ion batteries and filtration membranes.

Some methods have been brought forward to enhance deposition uniformity. Yang et al. used a conductive spherical-hat target as an electrospinning collector to change the electric field to achieve greatly improved uniformity [15]. Zheng et al. designed a two-step spinneret which can enhance the electric field density in the center area of the plate spinneret, and a curved collector which creates a more uniform surface electric field [16]. However, these methods are not suitable for large-area and roll-to-roll industrial production. Until now, the uniform deposition method of massive electrospinning has rarely been discussed and still remains a challenge.

In this paper, an auxiliary conductive electrode connected to the emitting electrode is introduced to Tip-Induced Electrospinning (TIE) to improve the deposition uniformity of nanofibers. TIE without and with three different auxiliary electrode shapes (plain, rectangular and boat-like) is chosen to explore the electric field distribution between electrodes, respectively, by finite element analysis (FEA) and the effects of shape and location of the auxiliary electrode on the morphologies of the nanofibers, the jet trajectory and the deposition uniformity, etc., are discussed. We aim to offer an easy and effective way to gain the uniform deposition of nanofibers in massive electrospinning.

## 2. Electric Field Simulation

**Figure 1:**
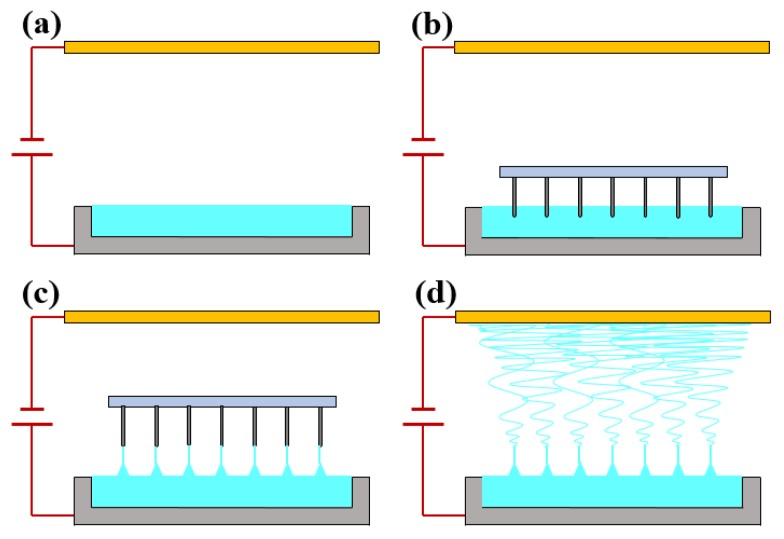
Schematic diagram of traditional Tip-Induced Electrospinning (TIE) setup.

**Figure 2:**
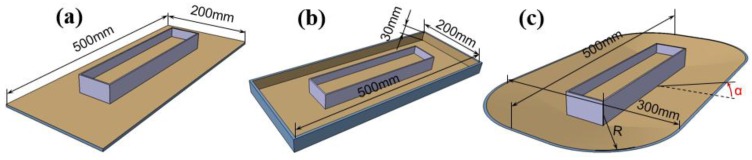
Different shaped diagrams of the conductive auxiliary electrodes around the solution reservoir: (**a**) plain; (**b**) rectangular circle; and (**c**) boat-like; the tilted angle is denoted by α.

The Tip-Induced Electrospinning process is presented in Figure 1a–d, in which tips are dipped into polymer solution and withdrawn quickly. Then Taylor cones can be formed from the solution surface where the tips left because of the adhesion of the polymer solution to the tips. After the tips move away, jets will come into being easily from the top of the Taylor cones with the help of the electric field force. The jets during the TIE process often travel to the surrounding area. Therefore, different shapes of auxiliary electrodes were designed to change the electric field nearby to control the trajectory of the jets, and the uniformity of the electric field was simulated by commercial FEA software to optimize the structure. The geometries of the FEA models were built according to the experimental setup and three different shapes of additional conductive electrode structures are shown in Figure 2. In all simulations, a voltage of 40 kV was applied to the metallic solution reservoir and the auxiliary electrode, and the collector was connected to the ground earth.

**Figure 3:**
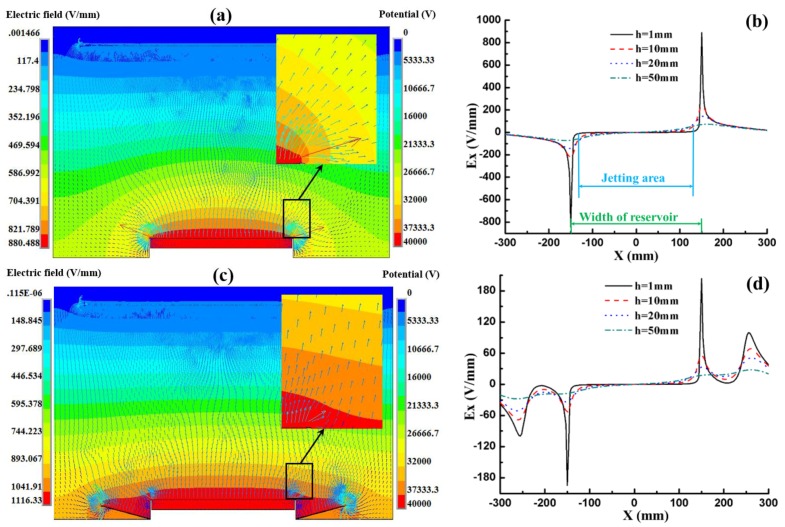
Electric field distribution without auxiliary electrode in (**a**) and (**b**) and with a boat-like electrode in (**c**) and (**d**); (b) and (d) show the *x* component electric field strength (absolute value) along the width of the reservoir with different heights above the solution surface.

The effect of the auxiliary electrode on the electric field was discussed at first by using a boat-like auxiliary electrode at the bottom of the solution reservoir with a 15° tilted angle. As depicted in Figure 3, the simulation results of the electric field distribution in TIE between the models with and without an auxiliary electrode are quite different. Figure 3a,b show that the maximum electric field along the *x*-axis (*E_x_*) in TIE without any auxiliary electrode at the heights of 1 mm, 10 mm, 20 mm and 50 mm above the solution surface is 890 V/mm, 229 V/mm, 141 V/mm and 70 V/mm, respectively, which are stronger but the electric field uniformity is poor. Figure 3c indicates that the introduction of an auxiliary electrode makes vector arrows near the edge of the reservoir become more vertical. In addition, the maximum *E_x_* at the corresponding height decreases to 203 V/mm, 53 V/mm, 33 V/mm and 18 V/m, respectively, as shown in Figure 3d. It can be concluded that the auxiliary electrode successfully decreases the maximum E_x_ and improves the electric field uniformity.

Since the auxiliary electrode has been proved to be effective in improving the distribution of the electric field, different shapes were designed and optimized to achieve the most uniform electric field. The electric field is expected to have a smaller *x* component (*E_x_*) and a more uniform *y* component (*E_y_*). Considering that the jetting area (from –120 mm to 120 mm along the *x*-axis) near the solution of the real setup is smaller than the width of the reservoir (from –150 mm to 150 mm along the *x*-axis), the E_x_ caused by the electric edge effect at a lower height (as shown Figure 3d, *h* = 1 mm, black curves) is nearly zero in the jetting area. Although the jetting area grows larger with the increasing height, the *E_x_* also becomes too weak to influence the jets. So the electric field at *h* = 10 mm was chosen to evaluate the electric field uniformity in different conditions.

**Figure 4:**
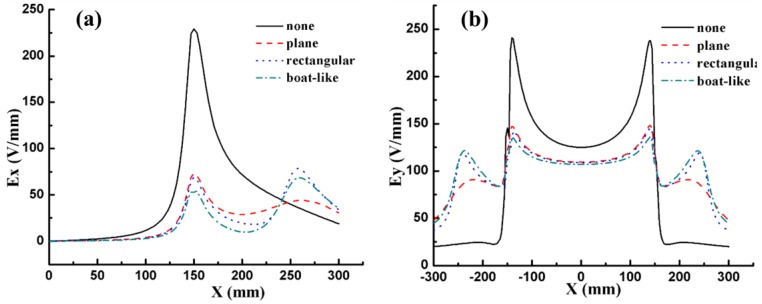
(**a**) *x* and (**b**) *y* component of electric field strength distribution at 10 mm above the solution surface with and without different shapes of auxiliary electrodes.

In view of the symmetry of the model, the electric field distribution in the positive direction was enough to express all the information about the electric field strength. *E_x_* and *E_y_* caused by different shapes are described in Figure 4. The simulation results illustrated that the maximum *E_x_* value was 229 V/mm, 74 V/mm, 70 V/mm and 53 V/mm for the TIE without the auxiliary electrode and with plain, rectangular and boat-like electrodes, respectively. It is obvious that the boat-like electrode resulted in a smaller E_x_ and a more uniform *E_y_* distribution at 10 mm above the solution surface (near the first peak). The plain auxiliary electrode causes a smaller *E_x_* beside the reservoir (the second peak), but it has little influence on the jets because this area is away from the jets’ trajectory. Thus, a boat-like auxiliary electrode is considered a better choice if only the electric field is considered.

**Figure 5:**
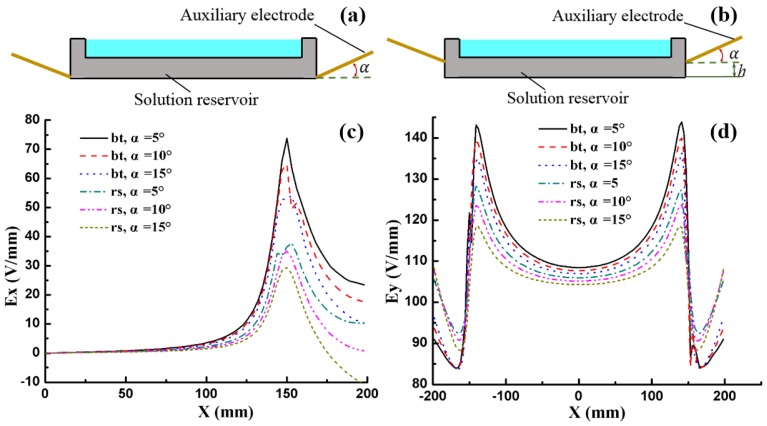
The electric field distribution for different tilted angles and different positions of the boat-like auxiliary electrode. The schematic diagram of the auxiliary electrode at the bottom of solution reservoir (**a**) and at the height 20 mm above the bottom (**b**); *E_x_* and *E_y_* distributions when the boat-like auxiliary electrode was (**c**) at the bottom and (**d**) at 20 mm above the reservoir bottom.

Furthermore, the tilted angle and the position of the boat-like auxiliary electrode also affected the electric field. As described in Figure 5, three different tilted angles (α= 5°, 10°, 15°) and two positions were set to study the best geometry. It indicated that if the boat-like auxiliary electrode was at the bottom of the reservoir (bt for short), the maximum *E_x_* was 74 V/mm, 65 V/mm and 52 V/mm for the tilted angles of 5°, 10° and 15,° respectively. When the auxiliary electrode was raised 20 mm above the bottom (rs, for short), the maximum *E_x_* can be reduced to 36 V/mm, 35 V/mm and 29 V/mm for the corresponding tilted angle. It is obvious that a larger tilted angle and higher position lead to a smaller *E_x_* and a more uniform *E_y_* distribution, respectively. However, *E_y_* was slightly reduced at the same time, which brings a negative effect for the formation of jets and nanofibers. A boat-like auxiliary electrode with a tilted angle 15° at 20 mm above the reservoir bottom seems to be the best choice according to the FEA simulation results. Whether it agrees with the experimental results was then demonstrated.

## 3. Experiments and Discussion

### 3.1. Materials and Methods

Poly (ethylene oxide) (PEO, *Mn* = 300,000, Huagao Jingxi Chemical, Changchun, China), which was inclined to spin at moderate potentials in direct current mode [17], was dissolved in the mixture of deionized water and ethanol with mass ratio of 3:1 to obtain the polymer solution of a concentration of 16 wt. %.

**Figure 6:**
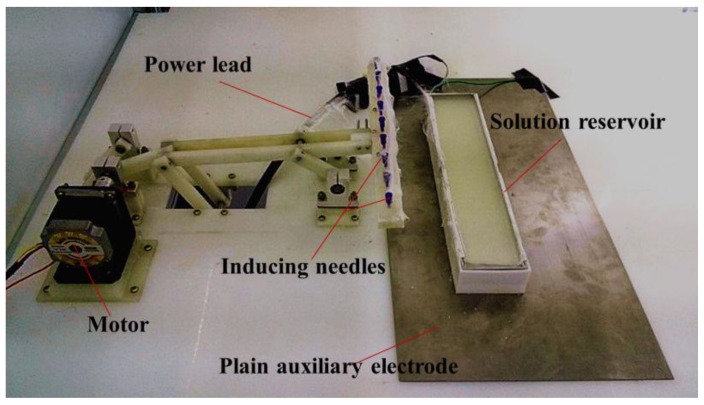
An optical photograph of TIE setup.

**Figure 7:**
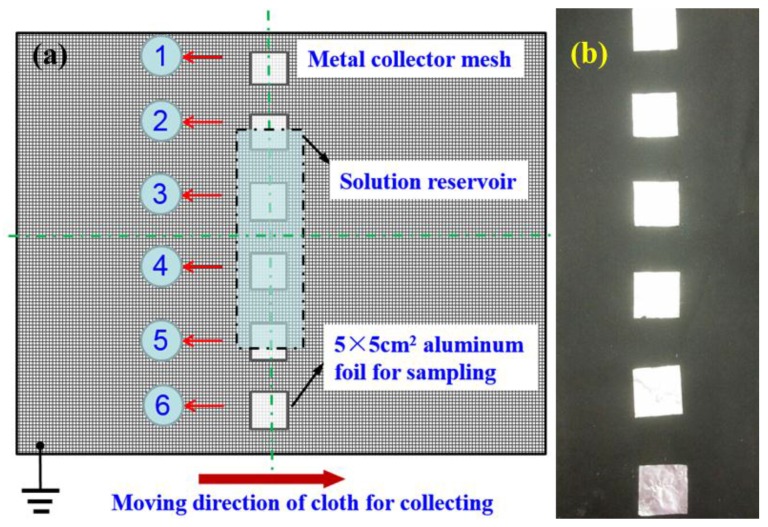
(**a**) Schematic diagram of six equally distributed samples on the collector. The collector is 60 cm in width. The arrow represents the moving direction of the collection cloth. (**b**) A photograph of the arrangement of the collector belt.

As seen in Figure 6, the TIE setup consists of a collector (not shown), an inducing component, a high-voltage source (only power lead was shown), a solution reservoir and an auxiliary electrode. The collector was composed of a grounded metal mesh, a long cloth to collect nanofibers and a winding device to realize continuous movement of the cloth. The inducing component contains a bar with arrayed inducing needles, which was driven by a motor through the crank and a bar linkage. The solution reservoir consists of a metal sheet and a plastic shell. Thereinto, the solution reservoir was 300 mm in length and 60 mm in width and the plastic shell was 20 mm in depth. The anode and cathode of the high voltage source (ES80P-20W/DDPM, Gamma, Ormond Beach, FL, USA) were respectively connected to the solution reservoir and the grounded metallic mesh. There are 13 inducing needles with 0.295 mm in diameter and 20 mm gap distance between needles along the inducing bar. Due to the continuous deposition in large-scale production process, the moving direction of the collector is perpendicular to the length of solution reservoir and the deposition uniformity of nanofibers does not need to be considered. In order to evaluate the deposition uniformity, as illustrated in Figure 7a, six 5 cm × 5 cm aluminum foils were evenly attached onto the collector, which width is 60 cm, in a line to receive PEO nanofibers. And Figure 7b shows the real arrangement of the collector belt.

Three different shapes of auxiliary electrodes connected to the solution reservoir were then made. PEO solution was held in the solution reservoir. The applied voltage was set to be 40 kV and the working distance between solution reservoir and grounded collector was 40 cm. The driving frequency of the inducing component was about 5/6 Hz and the electrospinning time was set to 15 min. During the experiments, the ambient temperature and humidity were kept around 23 °C and 40% RH respectively.

A digital camera (Canon D500, Canon INC, Tokyo, Japan) was used to record the trajectory of initial jet and the morphologies of PEO nanofibers were characterized by scanning electron microscopy (SU 70, Hitachi, Japan) after being sputtered with a thin layer of gold. The mass of all samples was measured by electronic balance (BS124, Sartorius Co., Göttingen, Germany) after drying at 50 °C for 30 min in the incubator.

### 3.2. Results and Discussion

**Figure 8:**
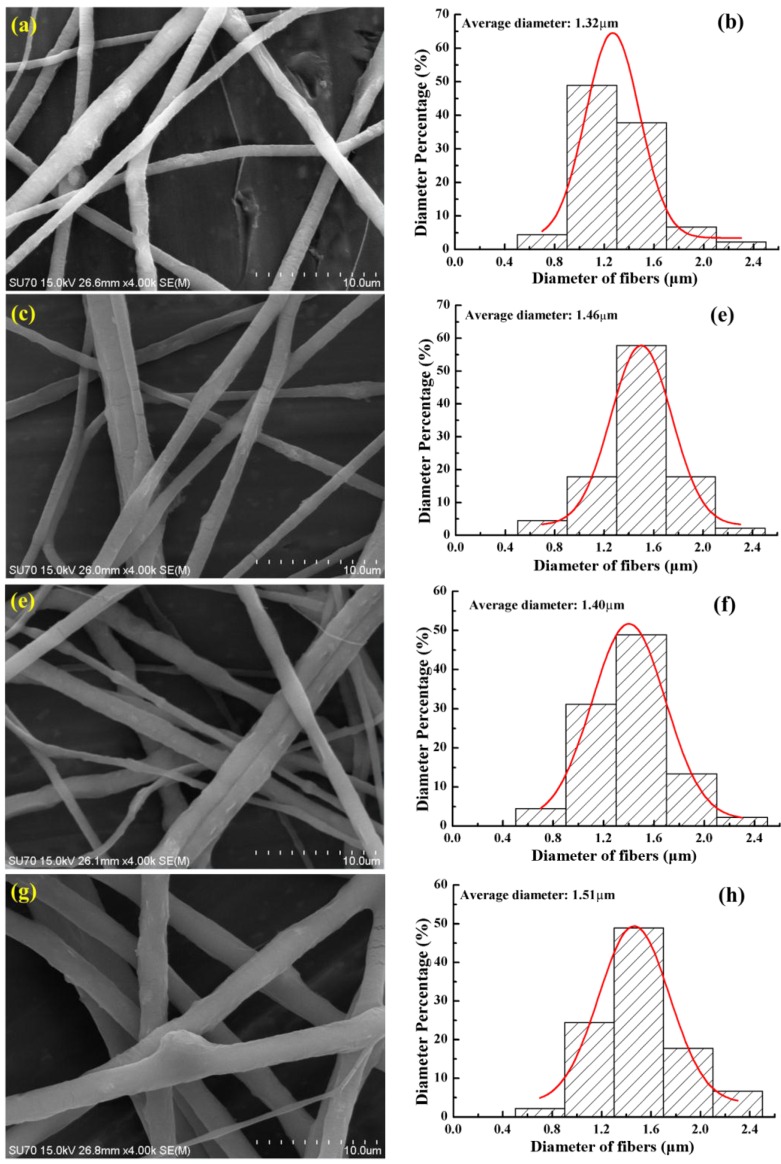
Scanning electron microscope (SEM) images and diameter distributions of poly (ethylene oxide) (PEO) fibers fabricated by TIE with different structures of the additional metal electrode: (**a**,**b**) none, (**c**,**d**) plain, (**e**,**f**) rectangular circle, (**g**,**h**) boat-like.

*Morphologies of fibers.* The effect of the auxiliary electrode shape on the morphologies of the electrospun PEO nanofibers by TIE was investigated without and with three different shapes (plain, rectangular and boat-like) of electrodes, respectively. From the scanning electron microscope (SEM) images in Figure 8, it can be seen that the diameter of the fibers varies in a range from 0.7 μm to 2.9 μm, and the mean diameter of the fibers from TIE without and with plain, rectangle and boat-like electrodes is 1.32 µm, 1.46 µm, 1.40 µm, and 1.51 µm, respectively. The diameter of the fibers from TIE without the additional electrode is the smallest and the largest mean diameter is from TIE with a boat-like electrode. Such a phenomenon is mainly attributed to the reduction of the electric field in the *y* direction with auxiliary electrodes as shown in Figure 4b, which decreases the electric pulling force on the as-spun fibers.

**Figure 9:**
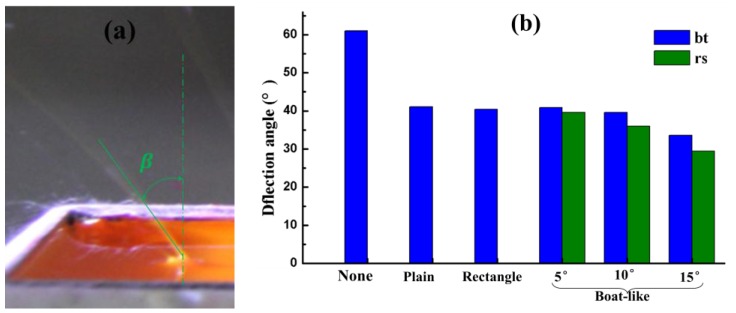
(**a**) The optical photograph of a typical deflection angle; (**b**) the deflection angle with respect to different shapes and positions of auxiliary metal electrodes.

*Initial jet trajectories.* The visible initial jet trajectories greatly affect the deposition uniformity of the nanofibers and they are characterized by the deflection angles of the jet at the edge of the reservoir from the *y*-axis because the deflection angle of the jets there is the biggest. If the deflection angle is zero, it means that the initial jets travel vertically and the nanofibers will be deposited evenly on the collector with a width of about 30 cm. As shown in Figure 9, when TIE was carried out with plain, rectangle and boat-like shaped electrodes with 5°, 10° and 15° tilted angles and without an auxiliary electrode, the deflection angles of the jets were 41.0°, 40.5°, 40.9°, 39.6°, 33.6°, and 61.0°, respectively. We can draw a conclusion that the boat-like electrode could reduce the reflection angle most greatly and the boat-like auxiliary electrode with a larger tilted angle will lead to a smaller β. Furthermore, when the boat-like auxiliary electrode was set at 20 mm above the bottom of the reservoir, the deflection angle could be decreased further to about 29.4°. Therefore, the minimum deflection angle could be achieved by using a boat-like electrode at 20 mm above the reservoir bottom with a tilted angle of 15°. This phenomenon is due to the reduction of E_x_ as illustrated in Figure 4a and Figure 5c. However, the initial deflection angle is only one of the factors that influence the deposition uniformity; therefore, a direct study of the deposition uniformity may be needed.

**Figure 10:**
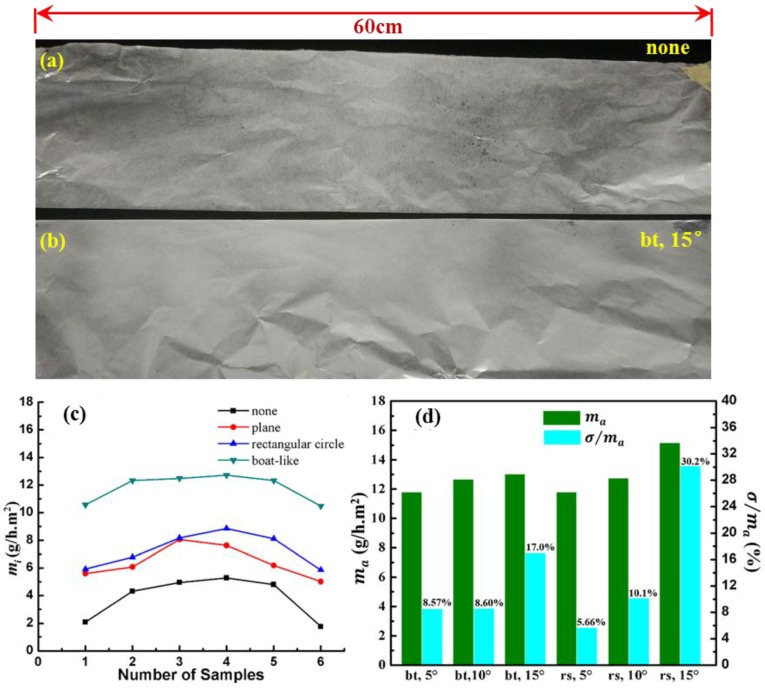
(**a**) The photograph of fibers on the continuous collector from TIE with no auxiliary electrode; (**b**) with a boat-like electrode with a 15° tilted angle electrode at the bottom. The length of the collector is 60 cm; (**c**) deposition efficiency distribution along *x*-axis for different shapes of auxiliary electrodes; (**d**) the average deposition efficiency and relative deposition deviation with different tilted angles and positions for boat-like electrodes.

*Uniformity of deposition.* We aim to find out the best way to improve the deposition uniformity of nanofibers. In order to examine whether it works in a real situation, all the fibers from TIE with no electrode or a boat-like electrode with a 15° tilted angle were shown in Figure 10a,b, respectively, which presented that the auxiliary electrode can effectively improve the deposition uniformity. To investigate the deposition uniformity from TIE with different auxiliary electrodes quantitatively, six pieces of 5 cm × 5 cm aluminum foils were attached onto the collector as shown in Figure 7 along the X-axis. Then the mass of nanofibers deposited on these samples within 15 min was measured to evaluate the uniformity of the deposition. The samples would contact the surface of an Al plate to release the static charges before being weighed in. The standard deviation of the mass of nanofibers on these samples was calculated by the following equation:

$$\sigma =\sqrt{\frac{1}{6}\overset{6}{\underset{i=1}{\sum }}{\left(m_{i}-m_{a}\right)}^{2}}$$

where σ is the standard deviation; *m_i_* (*i* = 1, 2, 3, ⋯ 6) represents the mass of nanofibers being collected on these different samples per hour in the unit area, which can be regarded as the deposition efficiency; *m_a_* is the average of all *m_i;_* and $\sigma /m_{a}$. was used to measure the deposition uniformity. Just as presented in Figure 10c, the mass in the center place is higher than that of the area. The average deposition efficiencies are 3.9, 6.4, 7.3 and 11.8 g/h⋅m^2^ from TIE without any additional electrode and with plane, rectangular and boat-like auxiliary electrodes. It is obvious that auxiliary electrodes can greatly increase the deposition efficiency of nanofibers to various degrees. Among these shapes, the boat-like auxiliary electrode can obtain the highest efficiency and the smoothest mass distribution of nanofibers. The increased deposition efficiency is attributed to the more vertical electric field distribution, which can make more fibers deposit onto the collector rather than travel to the side.

In order to determine the optimal geometry of the boat-like electrode, different tilted angles and positions of the boat-like electrode were then explored. As shown in Figure 10d, the average deposition efficiencies (*m_a_*) are 11.8, 12.7 and 13.0 g/h·m^2^ when the tilted angle is set as 5°, 10° and 15°, indicating that *m_a_* increases a little with the tilted angles. The boat-like electrode rose up with a height of 20 mm, and the average deposition efficiency is 11.8, 12.8, 15.2 g/h·m^2^ at the corresponding tilted angle. It should be attributed to the decrease of E_x_ as shown in Figure 5a, which allows more nanofibers to reach the collector. However, the relative deviation ($\sigma /m_{a}$) also increased greatly from 8.57% (5.66%) to 17% (30.2%) as the tilted angle increased from 5° to 15° at the “bt (rs)” position. Therefore, considering the deposition efficiency and the deposition uniformity, the optimal electrode structure is a boat-like shape with a tilted angle of 5° at 20 mm above the bottom of the reservoir, which can reduce the relative deviation from 39.9% with no auxiliary electrode to 5.66%. Thus, utilizing an auxiliary electrode can enhance the deposition uniformity greatly.

## 4. Conclusions

In this work, an auxiliary conductive electrode was introduced to the Tip-Induced Electrospinning process to improve the uniformity and efficiency of the deposition of fibers. Three different shapes of auxiliary electrodes were used to study their effect on the electric field uniformity, nanofiber diameter, jet deflection angle, deposition uniformity and efficiency. The simulation results show that the boat-like auxiliary electrode with a larger tilted angle most improved the uniformity of the electric field. Experimental results presented that the introduction of a boat-like auxiliary electrode at 20 mm above the reservoir bottom with a 5° tilted angle reduces the deflection angle of the jets from 61° to 29° and the production efficiency of the nanofibers on the collector can be enhanced from 3.87 to 11.8 g/h·m^2^. Therefore, the introduction of a boat-like auxiliary electrode to massive electrospinning may be a new method for improving the deposition uniformity of nanofibers.
